# Unusual presentation of hepatocellular carcinoma: Case report and review of literature

**DOI:** 10.1259/bjrcr.20210033

**Published:** 2021-04-09

**Authors:** Poonamjeet Loyal, Samuel Gitau, Soraiya Manji, Sitna Mwanzi, John Weru

**Affiliations:** 1 Department of Imaging and Diagnostic Radiology, Aga Khan University Hospital, Nairobi, Kenya; 2 Department of Imaging and Diagnostic Radiology, Aga Khan University Hospital, Nairobi, Kenya; 3 Department of Internal Medicine, Mediheal Hospital, Nairobi, Kenya; 4 Department of Hematology and Oncology, Aga Khan University Hospital, Nairobi, Kenya; 5 Department of Hemotology and Oncology, Aga Khan University Hospital, Nairobi, Kenya

## Abstract

Hepatocellular carcinoma (HCC) is the most common primary cancer of the liver and a major cause of mortality globally. Atypical presentation of HCC can present a diagnostic challenge. We, therefore, present a rare case of hepatocellular carcinoma fungating through the anterior abdominal wall with concomitant lung and brain metastases in a young patient with non-cirrhotic liver but positive chronic hepatitis B serology.

## Case presentation

A 26-year-old male presented with a two-year history of an anterior abdominal wall mass that was insidious in onset and progressively increasing in size. He had initially sought treatment at a rural facility for which we did not have access to the records or investigations done. When the patient presented to our facility, he had a fungating anterior abdominal wall mass but was otherwise well. While he was in the ward, he developed seizures. There was no history of alcohol intake.

## Investigations

**Figure 1.**
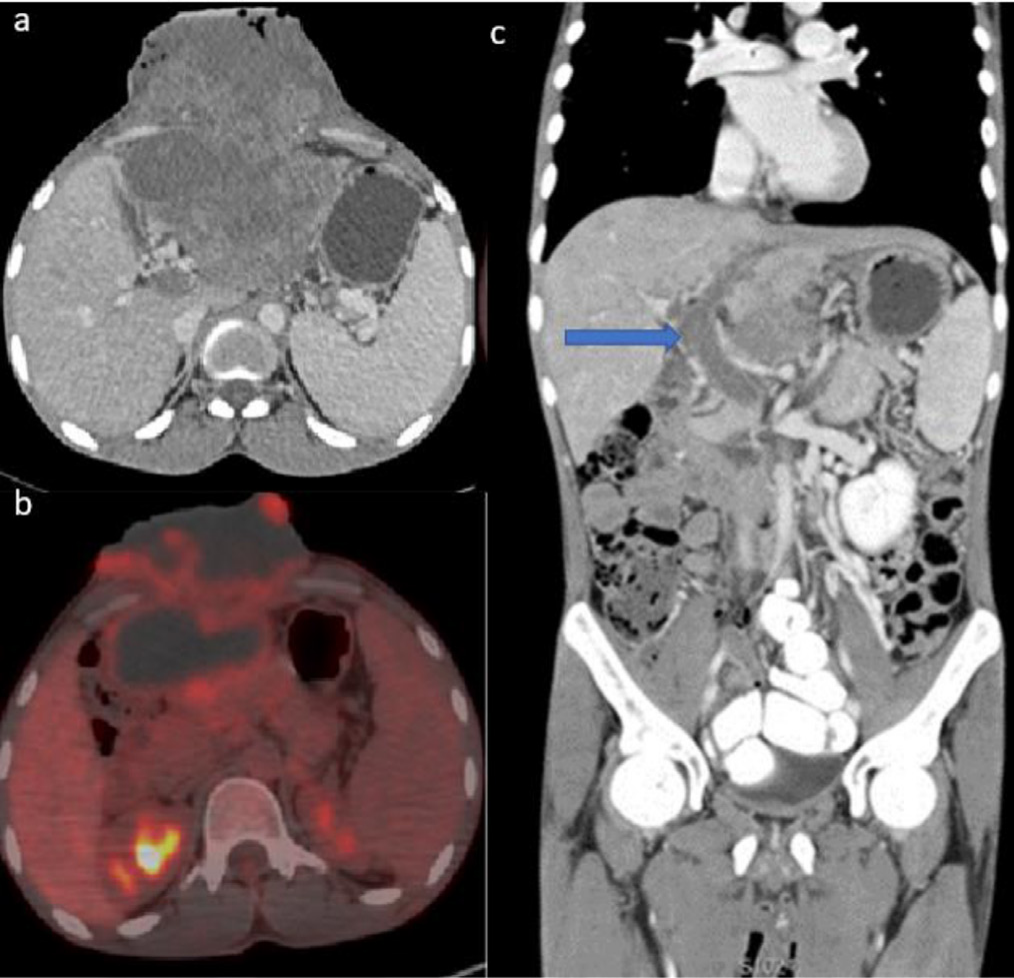
Axial porto-venous CT (a) and axial-fused FDG PET CT (b) images showing a large heterogenous left lobe of the liver mass invading the anterior abdominal wall. Coronal portovenous phase CT image (c) showing a thrombus in the main portal vein (arrow) and left portal vein.

**Figure 2.**
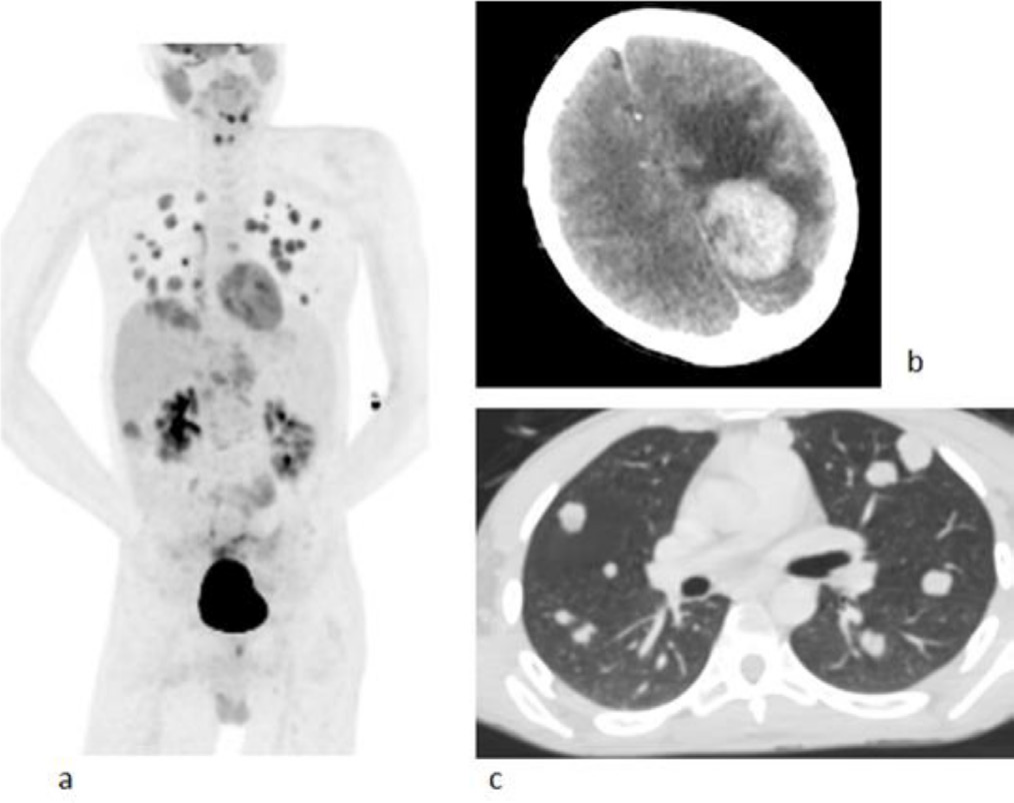
a. FDG PET MIP image showing satellites nodules in the liver and distribution of metastases in the lung; b. Axial non-enhanced CT of the head showing solitary hyperdense hemorrhagic metastases in the left parietal lobe; c. Axial non-enhanced CT of the chest showing the lung metastatic lesions.

Initial laboratory evaluation revealed a leukocytosis of 13 with a neutrophilia of 85. The liver function tests were also deranged up to five times higher than normal. The α fetoprotein was >50,000 and chronic hepatitis B serology was positive. HIV test was negative. As part of the initial evaluation, a CT chest and triphasic CT abdomen were performed followed by PET-CT to mainly look for other sites of distant metastases. Imaging revealed a large 16 × 15 × 13 cm heterogenous exophytic mass arising from the left lobe of the liver and invading the layers of the anterior abdominal wall muscles and the xiphisternum. No enhancement of the mass was seen in any of the phases. There was mild FDG uptake seen peripherally and satellite nodules in the rest of the liver. (Figure 1). Non-enhancing and non-FDG avid filling defect were present in the main portal vein, left portal vein with extension into the splenic vein and superior mesenteric vein, in keeping with thrombosis (Figure 1). There were no features of cirrhosis. No peritoneal nodes or abdominal or pelvis lymphadenopathy was seen. Additional sites of disease identified included direct infiltration of the xiphisternum, multiple lung nodules and a left parietal brain lesion (Figure 2).

**Figure 3.**
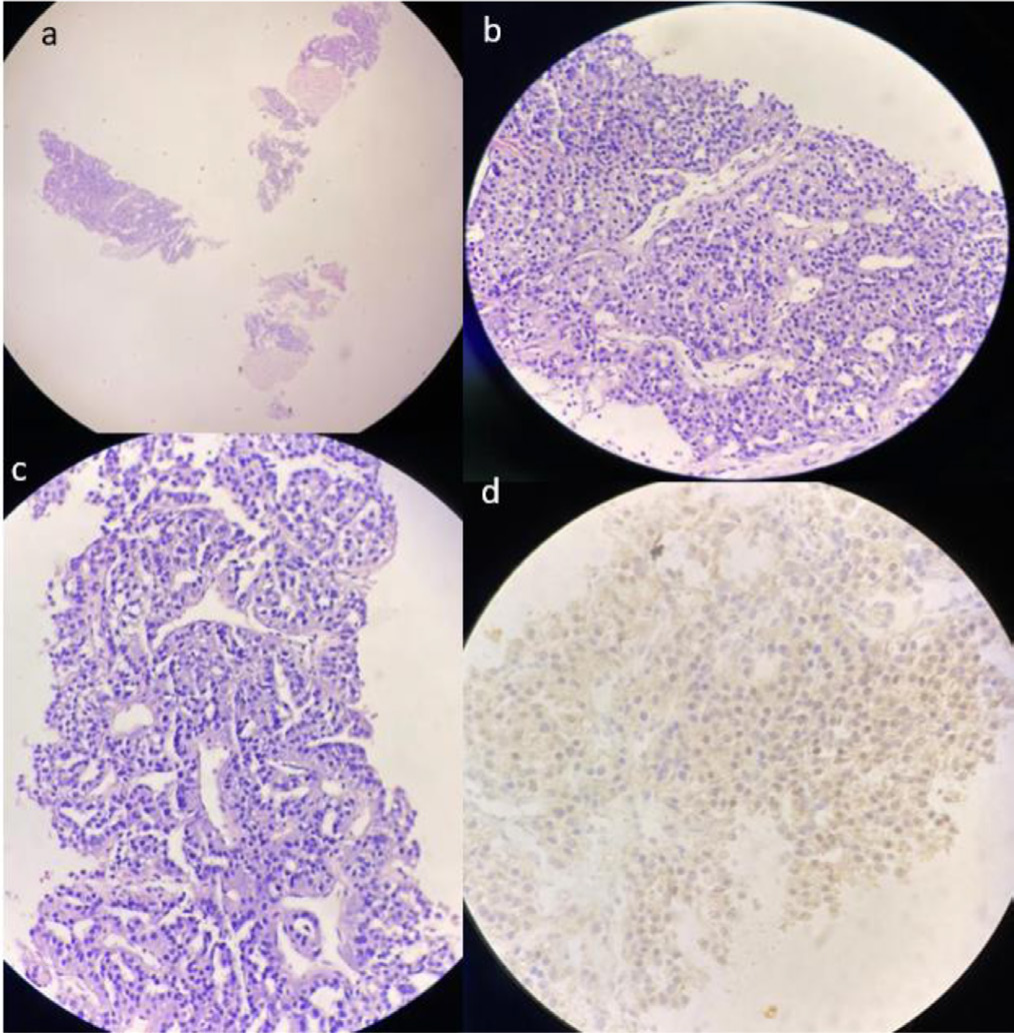
(a) Low power view with H&E staining showing no normal liver tissue, prominent fibrosis, and a diffuse tumor. High power views demonstrate intranuclear bodies (b) and endothelial wrapping (c), features suggestive of hepatocellular carcinoma. (d) Immunohistochemistry with Ttf1 shows nuclear dot-like staining confirming hepatocellular origin

Biopsy of the anterior abdominal wall mass revealed a tumor with solid nests and glands. Some nest showed prominent endothelial wrapping and no normal hepatocytes were seen. The cells showed round nuclei and abundant granular eosinophilic cytoplasm. Immunohistochemistry was consistent with hepatocellular carcinoma with CK-7 and CK 20 negative, TTF-1 positive and Hep par one negative (Figure 3).

## Treatment

The patient is currently under palliative care at our facility on sorafenib, levatinib, anti-epileptics and dexamethasone. Vacuum dressing with topical metronidazole constitutes the wound care treatment.*

Outcome and follow-up

The patient is currently able to make informed decisions and he continues with his daily activities and the seizures are also optimally controlled. Clinically, there has also been a decrease in size of the fungating component through the anterior abdominal wall.

## Discussion

This case highlights many atypical findings of HCC including young patient with a non-cirrhotic liver, large liver mass invading the anterior abdominal wall, a solitary hemorrhagic brain metastatic deposit, and multiple pulmonary metastases.

While hepatocellular carcinoma is associated with cirrhosis in 90% of the cases,^1^ it is rare in non-cirrhotic liver with a different clinical, treatment, and prognosis spectrum.^2,3^ HCC in non-cirrhotic liver tends not only to be larger (an average of 12.4 cm) and more heterogenous but it demonstrates a greater higher propensity for extrahepatic metastases at initial presentation^4^ just like in our case. This has been attributed to the lack of rigorous follow-up compared to those patients with cirrhosis.

In terms of demographics, it is twice more common in males with an average age at diagnosis is 65 years^5^ which differs from our case that presented very early at 26 years of age. This difference is likely due to the long-standing history of chronic hepatitis B infection in our patient which is a known predisposing factor for HCC development. To the best of our knowledge, there is only one reported case of hepatocellular carcinoma invading the muscles of the anterior abdominal wall which unlike our case spared the overlying skin with no distant metastases seen.^6^

Tumor thrombus is commonly associated with HCC and causes expansion and enhancement of the involved vessel.^7^ Bone metastases for HCC are invariably lytic while lung metastases are common occurring in up to 55% of patients consistent with our findings.^8^ Brain metastases are, however, very rare and intralesional hemorrhage is even rarer and often associated with a worse symptomatology and prognosis.^9^ In comparison with this, our patient, who had a solitary hemorrhagic metastasis and initially presented with convulsions, is, however, doing well currently.

## Learning points

1. Hepatocellular carcinoma is rare in patients with non-cirrhotic livers and when present may have an atypical presentation.
2. We found this case intriguing with a blend of rare and atypical features including a young patient with a non-cirrhotic liver, large liver mass invading the anterior abdominal wall, and a solitary hemorrhagic brain metastatic deposit.
3. Atypical presentation, like in our case, can delay treatment and we, therefore, aim to create awareness using this case.
